# Heparin Dosing Regimen Optimization in Veno-Arterial Extracorporeal Membrane Oxygenation: A Pharmacokinetic Analysis

**DOI:** 10.3390/pharmaceutics16060770

**Published:** 2024-06-06

**Authors:** Julien Lanoiselée, Jérémy Mourer, Marie Jungling, Serge Molliex, Lise Thellier, Julien Tabareau, Emmanuelle Jeanpierre, Emmanuel Robin, Sophie Susen, Benoit Tavernier, André Vincentelli, Edouard Ollier, Mouhamed Djahoum Moussa

**Affiliations:** 1 Department of Anesthesiology and Intensive Care Medicine, Saint-Etienne University Hospital, F-42055 Saint-Etienne, France; 2 INSERM U1059, Dysfonction Vasculaire et Hémostase, F-42055 Saint-Etienne, France; 3 Department of Anesthesiology and Intensive Care Medicine, CHU Lille, F-59000 Lille, France; 4 Department of Cardiac Surgery, CHU Lille, F-59000 Lille, France; 5 Inserm, CHU Lille, Institut Pasteur de Lille, U1011-EGID, University of Lille, F-59000 Lille, France; 6 ULR 2694-METRICS, Évaluation des Technologies de Santé et des Pratiques Médicales, University of Lille, F-59000 Lille, France; 7 Unité de Recherche Clinique Innovation et Pharmacologie, Saint-Etienne University Hospital, F-42270 Saint-Etienne, France

**Keywords:** heparin, extracorporeal membrane oxygenation (ECMO), pharmacokinetics, anticoagulation, anti-Xa

## Abstract

Background. Unfractionated heparin is administered in patients undergoing veno-arterial extracorporeal membrane oxygenation (VA-ECMO). Anticoagulation monitoring is recommended, with an anti-activated factor X (anti-Xa) targeting 0.3 to 0.7 IU/mL. Owing to heparin’s heterogeneous pharmacokinetic properties, anti-Xa is unpredictable, generating a challenge in anticoagulation practices. The aim of this study was to build a pharmacokinetic model of heparin accounting for potential confounders, and derive an optimized dosing regimen for a given anti-Xa target. Methods. Adult patients undergoing VA-ECMO were included between January 2020 and June 2021. Anticoagulation was managed with an initial 100 IU/kg heparin loading dose followed by a continuous infusion targeting 0.2 to 0.7 IU/mL anti-Xa. The data were split into model development and model validation cohorts. Statistical analysis was performed using a nonlinear mixed effects modeling population approach. Model-based simulations were performed to develop an optimized dosing regimen targeting the desired anti-Xa. Results. A total of 74 patients were included, with 1703 anti-Xa observations. A single-compartment model best fitted the data. Interpatient variability for distribution volume was best explained by body weight, C-reactive protein and ECMO indication (post-cardiotomy shock or medical cardiogenic shock), and interpatient variability for elimination clearance was best explained by serum creatinine and C-reactive protein. Simulations using the optimized regimen according to these covariates showed accurate anti-Xa target attainment. Conclusion. In adult patients on VA-ECMO, heparin’s effect increased with serum creatinine and medical indication, whereas it decreased with body weight and systemic inflammation. We propose an optimized dosing regimen accounting for key covariates, capable of accurately predicting a given anti-Xa target.

## 1. Introduction

Veno-arterial extracorporeal membrane oxygenation (VA-ECMO) is a temporary mechanical circulatory support procedure indicated for patients undergoing refractory cardiogenic shock or cardiac arrest [1]. Its use has increased over recent years, with almost 70,000 worldwide adult cases submitted to the Extracorporeal Life Support Organization (ELSO) registry [2]. Due to the contact between blood and nonendothelial surfaces of the circuit, VA-ECMO generates a coagulation activation mediated by the factor XII pathway, which also triggers an inflammatory reaction [3,4]. This phenomenon is associated with a high incidence of thrombotic complications that occur in more than 30% of cases [5]. To prevent these events, systemic anticoagulation is mandatory, and ELSO guidelines recommend the use of unfractionated heparin (UFH) as first-line therapy, mainly thanks to its short half-life and reversible effect with protamine [6]. On the other hand, VA-ECMO is also associated with a high incidence of bleeding complications that can be related to excessive anticoagulation [7].

Therefore, biological monitoring of the anticoagulation is crucial. The anti-factor X (anti-Xa) assay should be preferred over activated partial thromboplastin time (aPTT) due to its better association with UFH doses [8] and a lower sensitivity to preanalytical factors [9]. Although there is a paucity of evidence to relate anti-Xa with hemorrhagic or thrombotic events under VA-ECMO, a goal ranging from 0.3 to 0.7 UI/mL is recommended [6]. However, the administration regimen required to reach this biological target is unknown, resulting in a high variability in anticoagulation practices [10].

Indeed, UFH is a mixture of polysaccharides showing heterogeneous pharmacokinetic (PK) properties, generating unpredictable anti-Xa with a risk of over- or underdosing [11]. In a recent study, no relationship was demonstrated between the UFH dose and anti-Xa value under ECMO [8]. Interestingly, more than 2 days were needed to reach the targeted anti-Xa ranges, and several episodes of infratherapeutic targets were observed. These results underlined the need of a more reliable and predictable UFH administration strategy. Furthermore, the inflammation resulting from factor XII pathway activation, surgical insults, infections and critical illness may promote thrombin generation through tissue factor generation, endothelial cells and platelets activation, and thus affect UFH PK and pharmacodynamics (PD) [12,13]. To the best of our knowledge, this possible effect of inflammation on UFH PK has never been quantified nor considered in the establishment of a dosing regimen to reach the recommended anti-Xa target in VA-ECMO settings. The aim of this study was to develop a PK model accounting for factors influencing UFH PK exposure over time, including inflammation, in adult patients undergoing VA-ECMO, and infer an optimized dosing regimen.

## 2. Materials and Methods

### 2.1. Patients

This was a retrospective, observational, single-center study in which consecutive patients undergoing VA-ECMO at the Lille University Hospital Cardiac and Thoracic Intensive Care Unit (France) were included between January 2020 and June 2021. The exclusion criteria were as follows: duration of support less than 24 h, left ventricle unloading using Impella CP or 5.0 (due to the necessity of a supplemental UFH administration in our center) and outlier observations. The study was approved by the ethics committee of the French Society of Anesthesia and Intensive Care (IRB 00010254-2023-106) on 3 October 2023. Written informed consent was waived due to the retrospective design of the study.

### 2.2. Data Management

Patients’ characteristics, medical variables, biological observations and UFH administration data were extracted from our electronic patient’s management software (Sillage (SIB, Rennes, France, version V19), IntelliSpace Critical Care and Anaesthesia (Philips Healthcare, Koninklijke Philips N.V., Eindhoven, The Netherlands, version H.02.01)). According to usual care, the UFH doses were prospectively collected via a digital connection between electric syringes and electronic health records. The data were randomly split into model development and model validation cohorts. The development cohort was used to build the model and estimate population PK parameters. The validation cohort was used to evaluate the model predictiveness. Three of the authors (J.L., J.M. and E.O.) independently checked the extracted data.

### 2.3. Clinical Management

Patients were implanted with an extracorporeal circuit including a centrifugal pump (Rotaflow (Maquet Gentige group, Rastatt, Germany), Revolution (LivaNova Group, Saluggia, Italy), Cardiohelp (Maquet, Inc., Wayne, NJ, USA), ECMOLIFE (Euroset SPA, Medolla, Italy)) and a membrane oxygenator (Quadrox (Maquet Gentige group, Rastatt, Germany), Eos ECMO (LivaNova Group, Saluggia, Italy) and A.L.ONE ECMO oxygenator (Euroset, Medolla, Italy)) primed with crystalloid solution. Patients follow-up was ended at ECMO weaning, circuit change, necessity of antithrombin (AT) or protamine administration, or death.

### 2.4. Anticoagulation Management

Anticoagulation during VA-ECMO was managed with an initial 100 IU/kg loading dose of UFH before vascular cannulation, unless there was coagulopathy, post-cardiotomy or previous UFH administration (i.e., coronarography). Further UFH administration in the intensive care unit (ICU) was managed with a continuous intravenous electric infusion, adjusted to target an anti-Xa between 0.2 and 0.7 IU/mL according to patients’ medical conditions and ECMO status. Of note, UFH administration could be stopped, postponed or increased at physicians’ discretion in case of surgical reintervention, bleeding or thrombotic event, respectively.

### 2.5. Biological Sampling and Analysis

For PK analysis, UFH exposure was evaluated through an anti-Xa assay. Blood samples were collected regularly during ECMO support according to routine medical care, drawn in citrated tubes and analyzed on a STA-R Max analyzer (Diagnostica Stago, Asnière, France) using the same anti-Xa chromogenic assay, containing dextran sulfate without exogenous antithrombin (Biophen Heparin LRT, HYPHEN BioMed, Neuville-sur-Oise, France). The lower and upper limits of quantification were 0.1 IU/mL and 1.8 IU/mL, respectively.

### 2.6. Pharmacokinetic Model Development

The data were analyzed using Monolix modeling software (version 2023R1, Lixoft, Antony, France) with the SAEM algorithm, as previously described [14]. A population pharmacokinetic model was developed using a nonlinear mixed effects approach. Anti-Xa values were analyzed using the following framework:

*anti-Xa_ij_* = *F*(*t_ij_,ϕ_i_*) + (*a* + *b* × *F*(*t_ij_*,*ϕ_i_*)) × *ε_ij_*

where *anti-Xa_ij_* is the observed anti-Xa for patient *i* at time *j*. The function *F*(*t_ij_*,*ϕ_i_*) corresponds to the anti-Xa returned by the model for patient *i* at time *j* with the individual PK parameters *ϕ*. Parameters a and b are the constant and proportional parts of the error model with *ε_ij_*~N (0, 1).

For model development, we first identified the best structural model by testing 1- and 2-compartment PK models. Individual parameters were assumed to be log-normally distributed.

Secondly, covariate evaluation was performed by testing two types of covariates: (1) non-time dependent covariates using values that did not vary from the baseline during ECMO run (age, baseline total body weight (BW) and ECMO indication (post-cardiotomy or medical)); (2) time dependent covariates that varied during ECMO support and were regularly collected throughout UFH administration time course (inflammation, quantified using plasmatic C-reactive protein (CRP) and fibrinogen, serum creatinine (SCr) and the need for continuous renal replacement therapy (CRRT)). Covariates were added in the model using a stepwise procedure with forward inclusion and backward elimination, according to an algorithm performing simultaneous selection of the fixed and random effects, and based on the corrected Bayesian information criterion (BICc) whose penalties are adapted to mixed-effects models [15]. Covariates were kept in the model if they decreased the BICc and improved the goodness of fits as described below. Continuous covariates were log transformed, scaled to a typical value and tested with the following equation, using the effect of BW on distribution volume (*V*) as an example:

$$V_{i}=V_{POP}\times {\left(\frac{{BW}_{i}}{80}\right)}^{\theta_{BW}}\times e^{\eta_{i}}$$

where $V_{i}$ and ${BW}_{i}$ denote the individual values of *V* and *BW* for patient i, $V_{POP}$ is the typical value of parameter *V* estimated in the development cohort population, $\theta_{BW}$ is the estimated regression coefficient for *BW* on *V*, and $\eta_{i}$ is the random effect for the *i*-th patient, assumed to be normally distributed with a mean of zero and a variance equal to $\omega_{CL}^{2}$ (N [0, $\omega_{CL}^{2}$]). ${BW}_{i}$ is centered on 80 to provide an estimation of V for the typical BW value in the development cohort population.

Data below the lower limit of quantification and above the upper limit of quantification were left and right censored, respectively.

### 2.7. Pharmacokinetic Model Selection and Evaluation

Model selection was based on the decrease in corrected BICc and the visual inspection of the goodness of fit plots. Goodness of fit was obtained by plotting the observations versus population and individual predictions of the model, and the normalized prediction distribution errors (NPDE) versus time and population predictions [16]. Evaluation of the final model’s predictive properties was based on the visual inspection of the goodness of fit plots for the validation cohort using the parameters of the model. All figures and graphics were generated using the ggplot2 package with R software (version 4.1.1).

### 2.8. Individualized Regimen Estimation

Using the parameters estimated in the model and the significant covariates, we developed an individualized dosing regimen to achieve the desired anti-Xa target using an intravenous bolus loading dose followed by a continuous intravenous infusion maintenance dose, as previously described [17].

The loading dose (*LD*) was calculated as follows:

*LD* = *V_pop_* × *T_anti-Xa_*

where *V_pop_* corresponds to the covariate-adjusted typical value of the distribution volume and *T_anti-Xa_* corresponds to the target anti-Xa.

For calculation of the continuous infusion maintenance dose (*MD*), we used the following equation:

*MD* = *CL_pop_* × *T_anti-Xa_*

where *CL_pop_* corresponds to covariate-adjusted typical values of the clearance parameters.

### 2.9. PK Simulations

To evaluate the influence of the significant covariates on UFH exposure, and the ability of our optimized dosing regimen to reach the desired anti-Xa target, we performed PK simulations using the parameters estimated in our final model. Individual parameters values were sampled from the population distributions (n = 5000 simulations).

According to the parameter estimates (typical value and between subject’s variability) and the equations developed for the individualized regimen calculation, the interpatient variability of the doses required to achieve a target anti-Xa was estimated using the same simulation procedure. The unexplained variability and the effect of each covariate were graphically represented.

Simulations were generated using Mlxplore software (version 2019R2, Lixoft). Graphs of the results were obtained using the ggplot2 package with R software (version 4.1.1).

## 3. Results

### 3.1. Patients

During the screening period, 97 patients were implanted with VA-ECMO. A total of 74 patients were included in the study, 64 in the development cohort and 10 in the validation cohort. The patients’ characteristics are detailed in Table 1. The mean (±standard deviation) age was 52 (±13) years, the median (25th to 75th quartile) baseline total body weight (BW) was 75 (54–122) kg and the median ECMO duration was 7 days. A total of 1703 blood samples were collected for anti-Xa measurement (1487 in the development cohort and 216 in the validation cohort), in which 426 (25%) were left censored and 5 (0.3%) were right censored.

### 3.2. Pharmacokinetic Model

The best structural model to fit the data was a 1-compartment model, with a combined error model. Interpatient variability was estimated for parameters V and CL, corresponding to the volume of distribution and the elimination clearance, respectively.

After covariate inclusion, interpatient variability for V was best explained by BW, CRP and ECMO indication, and interpatient variability for CL was best explained by SCr and CRP. None of the other tested covariates significantly improved the fit. PK parameters estimates of the final model are displayed in Table 2. Inclusion of the significant covariates in the final model resulted in a 159.49-point reduction in BICc.

**Figure 1:**
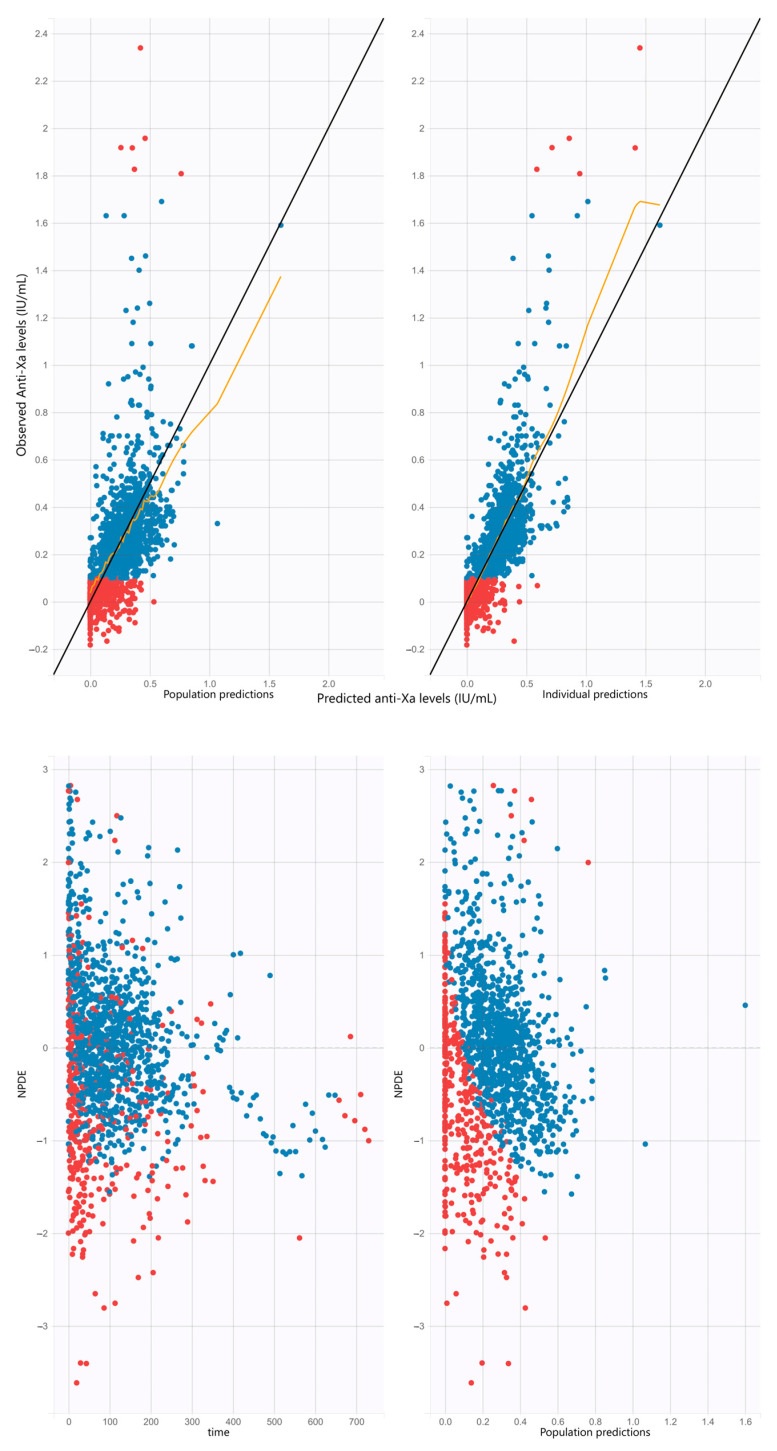
Goodness of fit plots for the development cohort. (**Top panels**): observations versus predictions. The black line represents the identity line. Blue circles represent the observed anti-Xa versus the corresponding predicted anti-Xa. Red circles represent the censored data. The yellow line represents the trend line. (**Left panel**): plot of the observed anti-Xa (IU/mL) versus population predicted (no random component). (**Right panel**): plot of the observed anti-Xa versus individual predicted anti-Xa (with random component). (**Bottom panels**): NPDE versus time and population predictions. The black line represents the identity line. NPDE, normalized prediction distribution errors.

Visual inspection of the goodness of fit plots for the development and validation cohorts showed no apparent bias in model predictions (Figure 1 and Figure S1, respectively).

### 3.3. Optimized Dosing Regimen

Using the parameters estimates of the final model with the formulae detailed above, we developed an individualized UFH dosing regimen adapted to BW, SCr, CRP and ECMO indication, with a loading dose and a maintenance dose calculated according to the following equations:

$${LD}_{i}={Vpop\times e}^{{Indic}_{i}\times \theta_{Indic}}\times {\left(\frac{{BW}_{i}}{80}\right)}^{\theta_{BW}}\times {\left(\frac{{CRP}_{i}}{100}\right)}^{\theta_{CRP}}\times T_{anti-Xa}$$

where ${LD}_{i}$ corresponds to the individual loading dose required to reach $T_{anti-Xa}$ for patient *i*; *V_pop_* represents the typical value of the distribution volume; *Indic_i_* represents the ECMO indication for patient *i*, Indic being equal to 1 for post-cardiotomy indication and 0 for medical indication; *BW_i_* and *CRP_i_* represent the individual values of BW and CRP for patient *i* scaled to the typical values of the development cohort population; and $\theta_{Indic}$, $\theta_{BW}$ and $\theta_{CRP}$ represent the regression coefficients of ECMO indication, BW and CRP on *V*, respectively.

$${MD}_{i}=CLpop\times {\left(\frac{{SCr}_{i}}{115}\right)}^{\theta_{SCr}}\times {\left(\frac{{CRP}_{i}}{100}\right)}^{\theta_{CRP}}\times T_{anti-Xa}$$

where ${MD}_{i}$ corresponds to the individual maintenance dose required to reach $T_{anti-Xa}$ for patient *i*; *CL_pop_* represents the typical value of the elimination clearance; *SCr_i_* and *CRP_i_* represent the individual values of *SCr* and *CRP* for patient *i* scaled to the typical values of the development cohort population; and $\theta_{SCr}$ and $\theta_{CRP}$ represent the regression coefficients of *SCr* and *CRP* on *CL*, respectively.

**Figure 2:**
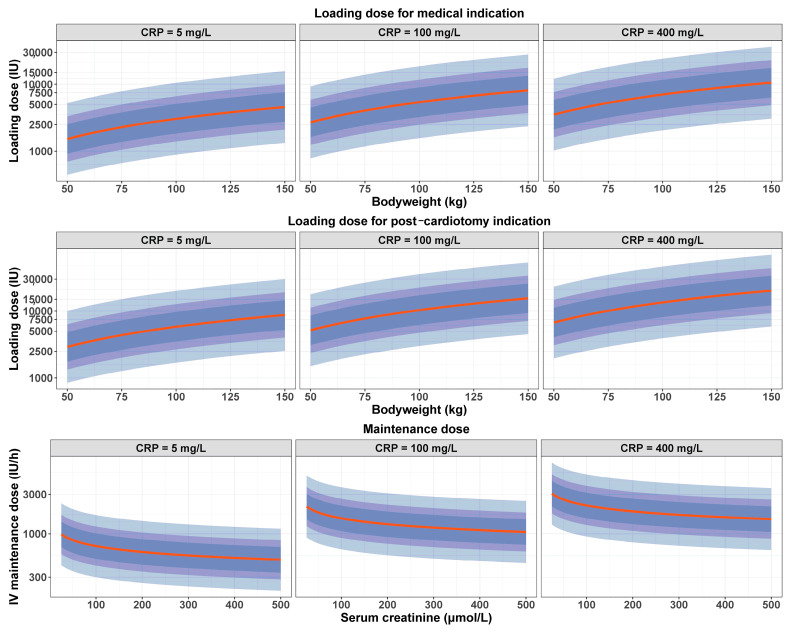
Optimized dosing regimen estimated to reach a 0.5 IU/mL target anti-Xa. Loading dose depended on body weight, CRP and ECMO indication ((**top row**): medical indication, (**middle row**): post-cardiotomy indication). Continuous IV (intravenous) maintenance dose (**bottom row**) depended on serum creatinine and CRP. (**Left column**): CRP 5 mg/L. (**Middle column**): CRP 100 mg/L. (**Right column**): CRP 400 mg/L. Red line represents the median dose to reach the target. Dark, average and light blue shaded areas correspond to the interpatient variability intervals estimated in our model (50%, 70% and 90%, respectively). The loading dose increased with increasing body weight and CRP, and with post-cardiotomy indication. The maintenance dose decreased with renal impairment and decreasing CRP.

Figure 2 displays the graphs of the optimized UFH dosing regimen required to target a 0.5 IU/mL anti-Xa using an LD depending on BW, CRP and ECMO indication (top row: medical indication, middle row: post-cardiotomy indication), and an MD depending on SCr and CRP (bottom row). The loading dose increased with increasing BW and CRP, and with post-cardiotomy indication. The maintenance dose decreased with renal impairment and decreasing CRP.

### 3.4. PK Simulations

**Figure 3:**
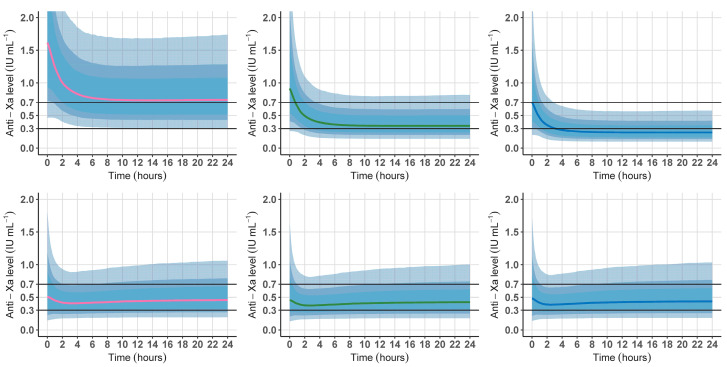
Simulations of the anti-Xa time courses using the final PK model with focusing on CRP. Simulations were performed for a medical patient weighing 80 kg with a serum creatinine of 115 µmol/L. Red line: CRP 5 mg/L. Green line: CRP 100 mg/L. Blue line: CRP 400 mg/L. The black lines correspond to the recommended 0.3–0.7 IU/mL target anti-Xa interval. Dark, average and light blue shaded areas correspond to the interpatient variability intervals estimated in our model (50%, 70% and 90%, respectively). (**Top panels**): simulations according to an 8000 IU (100 IU/kg) loading dose immediately followed by a 1200 IU/h (15 IU/kg/h) without adaptation to body weight or renal function. (**Bottom panels**): simulations according to our optimized dosing regimen. (**Bottom left panel**): 2500 IU loading dose followed by a 750 IU/h maintenance dose. (**Bottom middle panel**): 4000 IU loading dose followed by a 1500 IU/h maintenance dose. (**Bottom right panel**): 5500 IU loading dose followed by a 2200 IU/h maintenance dose.

To quantify the influence of inflammation on UFH exposure, simulations were first performed using a standard UFH dose for a typical patient with varying covariates. Figure 3 (top panels) displays anti-Xa time courses simulated with our model for a medical patient weighing 80 kg with an SCr of 115 µmol/L. The UFH dosing regimen was simulated according to an 8000 IU (100 IU/kg) loading dose immediately followed by a 1200 IU/h (15 IU/kg/h) continuous infusion during 24 h, without adaptation to renal function or CRP. For a 100 mg/L CRP (typical value in the development cohort, top middle panel), visual inspection of the simulated data showed that anti-Xa were included within the 0.3–0.7 IU/mL interval during the whole administration time course. For a CRP equal to 5 mg/L (top left panel), anti-Xa were above 0.7 IU/mL. For a CRP equal to 400 mg/L, anti-Xa were mostly below the 0.3 IU/mL threshold (top right panel).

PK simulations were secondly performed with the same covariates using our optimized dosing regimen (Figure 3, bottom panels). Graphs of the results showed that the 50% confidence intervals of the simulated anti-Xa values were included within the 0.3–0.7 IU/mL target interval during the whole administration duration. Similar simulations were performed for ECMO indication, BW and SCr, and are shown in Supplementary Figure S2–S4.

## 4. Discussion

This study provides new insights into the PK of UFH in adult patients undergoing VA-ECMO. Renal function, BW, inflammation and ECMO indication (post-cardiotomy or medical) were identified as significant covariates explaining UFH exposure variability. Using our model, we developed an optimized dosing regimen capable of achieving the desired anti-Xa, and we performed PK simulations to evaluate the impact of relevant covariates on the UFH dosing scheme. Among these covariates, our simulations showed a decrease in UFH exposure with increased inflammation, depicting the need to increase doses to maintain therapeutic anticoagulation.

To our knowledge, this is the first study to perform a PK analysis of UFH in adult VA-ECMO patients using a nonlinear mixed-effects modeling approach. Among the previous studies that investigated UFH PK [18,19,20,21,22,23], only a few focused on cardiopulmonary bypass (CPB) [21,22] or ECMO [23]. Similar analyses were performed in adult patients undergoing CPB for cardiac surgery, using either anti-activated factor II (anti-IIa) [21] or anti-Xa [22] assays. In both studies, UFH exposure was best described by a 2-compartment model. No covariate was identified to improve the fit in one study [21], whereas BW was found to affect central V and CL in the other [22]. However, this evidence obtained in the setting of CPB cannot be translated to VA-ECMO due to the fundamental differences observed between these two supports.

The single study available on ECMO before our research included exclusively pediatric patients and was undermined by the heterogeneity of the population characteristics, which consisted in both veno-venous (VV) and VA-ECMO [23]. This study was performed using real-world data collected retrospectively, with anti-Xa routinely measured to describe UFH PK. A total of 159 patients were included and 2140 observations were analyzed. Contrarily to our study, UFH administration data were not recorded prospectively as part of standard care, and some data were missing due to the retrospective design, which could have increased collection bias. Statistical analysis was performed using a method similar to our study. However, observations below the limit of quantification were treated as missing values, whereas they were handled as censored data in our study to decrease bias and information loss. As in our results, UFH exposure was explained by a 1-compartment model with a combined error model. The population parameters were similar with our model. Concerning the covariates analysis, BW had a significant effect on both V and CL, as in our model. Circuit change, performed at the discretion of the clinical team on the basis of clot burden within the circuit and oxygenator, had a significant effect on CL. However, this covariate does not seem to be relevant for UFH dosing individualization in clinical practice. Moreover, the authors did not investigate the effect of SCr, inflammation and ECMO indication on UFH PK, and these data may not be extrapolated to our population, as pediatric patients are known to present specific PK characteristics [24,25,26].

In the current study, several covariates were identified to explain UFH PK variability. Firstly, baseline BW was acknowledged to explain interpatient variability for V, confirming existing literature describing the need to individualize the UFH loading dose according to BW in CPB or ECMO [22,23]. Secondly, SCr, a surrogate of glomerular filtration capabilities or CRRT filtration performance, influenced UFH exposure. This finding underlines the role of kidney function in UFH clearance through a slow but unsaturable and dose-independent renal mechanism, along with the rapid but saturable and dose-dependent reticuloendothelial system-dependent mechanism [11]. Although the role of renal route on heparin clearance is already known, our study is the first to underline this mechanism in the setting of VA-ECMO support, along with quantifying its influence on heparin exposure to propose an individualized dosing regimen adapted to renal function. Thirdly, systemic inflammation modulation of UFH effects is actually supported by physiological driven knowledge. UFH is known to bind to a various number of acute phase proteins, activated endothelial cells and macrophages, which are involved in the inflammatory response encountered in critically ill patients [12]. This phenomenon is considered among mechanisms involved in heparin resistance [27]. Yet, the VA-ECMO circuit may promote inflammation through a contact pathway additional to patient-related sources, which is associated with increased inflammation and a prothrombotic phenotype [28]. Further to the recent technological advances in biomedical engineering concerning biomimetic and biopassive surfaces, along with the development of endothelialized surfaces, clinical practice regarding anticoagulation in ECMO is still based on the large use of UFH, pending future goals that will feature biocompatible and bio-hybrid materials not requiring combined systemic anticoagulation [29]. Concerning circuit-induced inflammation, PK simulations regarding the CRP level showed that the anti-Xa effect of UFH for a given dose decreased with increasing CRP, with a risk of underdosing (Figure 3), confirming previous data in pediatric patients without ECMO depicting the need to increase UFH doses in the presence of inflammation [30].

Finally, concerning covariates effect, our study identifies VA-ECMO indication as a factor influencing UFH exposure. Post-cardiotomy patients presented a higher volume of distribution. These data could be explained by the exposition to CPB before VA-ECMO in post-cardiotomy patients, leading to fluid loading and hemodilution. In total, our optimized dosing regimen achieved the desired anti-Xa target considering these four significant covariates (Figure 3 and Figures S2–S4).

In this research, estimation of the PK parameters showed a residual interpatient variability, particularly for V (Figure 3 and Figures S2–S4). This could be attributed to bias encountered in our study. Firstly, the retrospective design provides a risk of imprecision in PK parameters estimation. Secondly, the UFH administration pattern with prolonged continuous infusion and few boluses was challenging for V estimation. However, interpatient variability for V and CL in our final model was lower than in Salem’s study [23] (75.3 versus 262.9% and 52.4 versus 58.9%, respectively). The residual variability could also be explained by unidentified factors or those known to interact with UFH, among which are antithrombin [11] or Von Willebrand factor [31]. Lastly, a large proportion of observations were censored, as they were out of the limits of quantification range, but this was considered in our statistical analysis. However, despite this residual variability, Figure 3 and Figures S2–S4 show that the proportion of over- or underdosing using our optimized dosing regimen (bottom panels) was lower than with the current dosing scheme used in our institution (top panels). Moreover, simulations using our optimized dosing regimen targeting a 0.5 IU/mL anti-Xa showed that the 50% confidence intervals of the simulations were included within the 0.3–0.7 IU/mL recommended interval. Evaluation of the model performance showed no apparent bias in model predictions (Figure 1 and Figure S1).

Another limitation of our study is the use of an anti-Xa chromogenic test, which is sensitive to plasmatic free hemoglobin, triglyceride and bilirubin [32]. However, the anti-Xa assay by protamine titration is not routinely available, and may undermine the clinical relevance of the final model. The other tests routinely used for UFH monitoring (activated clotting time and activated prothrombin partial time) are more prone to various preanalytical biases [8,33,34].

The use of SCr may be questionable, but remains the more widely available biomarker for GFR (glomerular filtration rate) evaluation. None of the GFR evaluation equations are validated in ICU patients, and particularly in the ECMO setting. Furthermore, variation of creatinine allows estimation of both native kidney function and CRRT performance in our development cohort, where 46% experienced a CRRT during ECMO course [35].

Despite these limitations, our study answered a major question regarding UFH administration during VA-ECMO. Setting an optimal anticoagulation dosing scheme to reach a defined target is a major issue, and results in large variations in medical practices [10]. During the inclusion period of our study, 43% of the observed data in the development cohort indicate underdosing with an anti-Xa value < 0.2 IU/mL. Our optimized dosing regimen may help clinicians to reach the desired target anti-Xa depending on the patient and ECMO conditions. This study did not intend to evaluate the association between UFH administration and bleeding or thrombotic complications, an issue which remains unsettled [8]. Further multicenter studies are needed to validate our PK model and perform PK-pharmacodynamic analysis.

In conclusion, we built a PK model of UFH in adult patients undergoing VA-ECMO and identified weight, renal function, CRP and ECMO indication as factors explaining UFH exposure variability. We developed an optimized dosing regimen able to reach the desired target anti-Xa according to these factors.

## Figures and Tables

**Table 1:** Patients’ characteristics.

	Total Population	Development Cohort	Validation Cohort
	n = 74	n = 64	n = 10
Age (years)	52 ± 13	51 ± 12	53 ± 15
Male gender	53 (71.6)	44 (68.8)	9 (90)
Total body weight (kg)	75 (54–122)	75 (54–122)	67 (56–105)
Comorbidities			
Diabetes mellitus	13 (17.6)	12 (18.8)	1 (10)
Chronic kidney disease	17 (23)	16 (25)	1 (10)
Strokes	2 (2.7)	2 (3.1)	0 (0)
Ischemic cardiopathy	16 (21.6)	15 (23.4)	1 (10)
Hypertension	16 (21.6)	15 (23.4)	1 (10)
Hypercholesterolemia	16 (21.6)	12 (18.7)	4 (40)
Atrial fibrillation	10 (13.5)	9 (14.1)	1 (10)
P2Y12 inhibitors during ECMO	16 (21.6)	12 (18.7)	4 (40)
Lactate on admission (mmol/L)	5 (1–23) ^a^	5.1 (1–23) ^a^	5.1 (1.7–12.4)
CRRT during ECMO	34 (45.9)	29 (45.3)	5 (50)
VA-ECMO indication			
PC-LCOS	29 (39.2)	26 (40.6)	3 (30)
Myocardial infarction	24 (32.4)	19 (29.7)	5 (50)
Myocarditis	4 (5.4)	3 (4.7)	1 (10)
Acute on chronic heart disease	6 (8.1)	5 (7.8)	1 (10)
Others ^b^	11 (14.9)	11 (17.2)	0 (0)
Description of VA-ECMO support			
ECMO duration (days)	7 (1–30)	7 (1–30)	7.5 (2–18)
Cumulative ECMO duration (days)	573	495	78
Peripheral ECMO	70 (94.6)	60 (93.7)	10 (100)
Mean UFH dose (IU/kg/h)	11.39	11.35	11.64
Weaning categories			
Successful weaning	45 (60.8)	39 (61.0)	6 (60)
Heart transplantation	5 (6.8)	5 (7.8)	0 (0)
Left or bi-ventricular assist devices	3 (4.0)	2 (3.1)	1 (10)
Death under ECMO	21 (28.4)	18 (28.1)	3 (30)
Covariates during VA-ECMO			
C-reactive protein (mg/L)	107 (3–547)	113 (3–547)	86 (7–345)
Fibrinogen (g/L)	4 (1–11.1)	5 (1–10)	3.7 (1.4–11.1)
Serum creatinine (µmol/L)	114.9 (26.5–530.4)	114.9 (26.5–530.4)	88.4 (53–415.5)

Values are expressed in numbers (percentage), mean ± standard deviation or median (range). VA-ECMO, veno-arterial extracorporeal membrane oxygenation; CRRT, continuous renal replacement therapy; PC-LCOS, post-cardiotomy low cardiac output syndrome; UFH, unfractionated heparin. ^a^ 2 missing data. ^b^ 6 cardiac arrests unrelated to myocardial infarction, 1 septic cardiomyopathy, 1 drug intoxication, 1 cardiogenic shock following transcatheter mitral valve replacement, 2 cardiogenic shocks from unknown etiology.

**Table 2:** Estimates of population parameters in the final model.

Parameter	Estimate (% RSE)
CL (L/h) = θ1 × (Scr/115)^θ2^ × (CRP/100)^θ3^	
θ1	3.41 (7.04)
θ2	−0.237 (0.18)
θ3	0.258 (0.66)
V (L) = θ4^Indic×θ5^ × (BW/80)^θ6^ × (CRP/110)^θ7^	
θ4	8.65 (15.7)
θ5	0.647
θ6	1 (fixed)
θ7	0.191 (0.45)
Ω_CL_ (SD)	0.52 (10.3)
Ω_V_ (SD)	0.75 (13.2)
BICc	−432.52
Additive residual variability (SD)	0.07 (5.42)
Proportional residual variability (SD)	0.41 (3.62)

RSE, relative standard error; CL, clearance; SCr, serum creatinine (µmol/L); CRP, C-reactive protein (mg/L); V, volume of distribution; Indic, ECMO indication, Indic being equal to 1 for post-cardiotomy indication and 0 for medical indication; BW, body weight; Ω, random effect variance for each parameter; SD, standard deviation; BICc, corrected Bayesian information criterion.

## Data Availability

The data presented in this study are available on request from the corresponding author due to legal reasons.

## Supplementary Materials

The following supporting information can be downloaded at: https://www.mdpi.com/article/10.3390/pharmaceutics16060770/s1. Figure S1. Goodness of fit plots for the validation cohort. Top panels: observations versus predictions. The black line represents the identity line. Blue circles represent the observed anti-Xa versus the corresponding predicted anti-Xa. Red circles represent the censored data. The yellow line represents the trend line. Left panel: plot of the observed anti-Xa (IU/mL) versus population predicted anti-Xa (no random component). Right panel: plot of the observed anti-Xa versus individual predicted anti-Xa (with random component). Bottom panels: NPDE versus time and population predictions. The black line represents the identity line. NPDE, normalized prediction distribution errors. Figure S2. Simulations of the anti-Xa time courses using the final PK model with focusing on ECMO indication. Simulations were performed for a patient weighing 80 kg with a serum creatinine of 115 µmol/L and a CRP of 100 mg/L. Green line: Medical indication. Red line: Post cardiotomy indication. The black lines correspond to the recommended 0.3–0.7 IU/mL target anti-Xa interval. Blue shaded areas correspond to the interpatient variability intervals estimated in our model (50%, 70% and 90%, respectively). Top panels: simulations according to an 8000 IU (100 IU/kg) loading dose immediately followed by a 1200 IU/h (15 IU/kg/h) without adaptation to body weight, renal function, or CRP. Bottom panels: simulations according to our optimized dosing regimen. Bottom left panel: 4000 IU loading dose followed by a 1500 IU/h maintenance dose. Bottom right panel: 8000 IU loading dose followed by a 1500 IU/h maintenance dose. Figure S3. Simulations of the anti-Xa time courses using the final PK model with focusing on BW. Simulations were performed for a medical patient with a serum creatinine of 115 µmol/L and a CRP of 100 mg/L. Red line: 50 kg. Green line: 80 kg. Blue line: 120 kg. The black lines correspond to the recommended 0.3–0.7 IU/mL target anti-Xa interval. Blue shaded areas correspond to the interpatient variability intervals estimated in our model (50%, 70% and 90%, respectively). Top panels: simulations according to an 8000 IU (100 IU/kg) loading dose immediately followed by a 1200 IU/h (15 IU/kg/h) without adaptation to CRP or renal function. Bottom panels: simulations according to our optimized dosing regimen. Bottom left panel: 3000 IU loading dose followed by a 1500 IU/h maintenance dose. Bottom middle panel: 4000 IU loading dose followed by a 1500 IU/h maintenance dose. Bottom right panel: 7000 IU loading dose followed by a 1500 IU/h maintenance dose. Figure S4. Simulations of the anti-Xa time courses using the final PK model with focusing on SCr. Simulations were performed for a medical patient weighing 80 kg with a CRP of 100 mg/L. Red line: SCr 25 µmol/L. Green line: SCr 115 µmol/L. Blue line: SCr 500 µmol/L. The black lines correspond to the recommended 0.3–0.7 IU/mL target anti-Xa interval. Blue shaded areas correspond to the interpatient variability intervals estimated in our model (50%, 70% and 90%, respectively). Top panels: simulations according to an 8000 IU (100 IU/kg) loading dose immediately followed by a 1200 IU/h (15 IU/kg/h) without adaptation to body weight or CRP. Bottom panels: simulations according to our optimized dosing regimen. Bottom left panel: 4000 IU loading dose followed by a 2000 IU/h maintenance dose. Bottom middle panel: 4000 IU loading dose followed by a 1500 IU/h maintenance dose. Bottom right panel: 4000 IU loading dose followed by a 1000 IU/h maintenance dose.
