# Supplementary material for: Heparin Dosing Regimen Optimization in Veno-Arterial Extracorporeal Membrane Oxygenation: A Pharmacokinetic Analysis
Source: Pharmaceutics. 2024 Jun 6;16(6):770. doi: 10.3390/pharmaceutics16060770 (PMC11207075; doi:10.3390/pharmaceutics16060770)
Supplement: Supplementary file 1 [file pharmaceutics-16-00770-s001.zip › pharmaceutics-2997215-supplementary.pdf]

Supplementary Materials

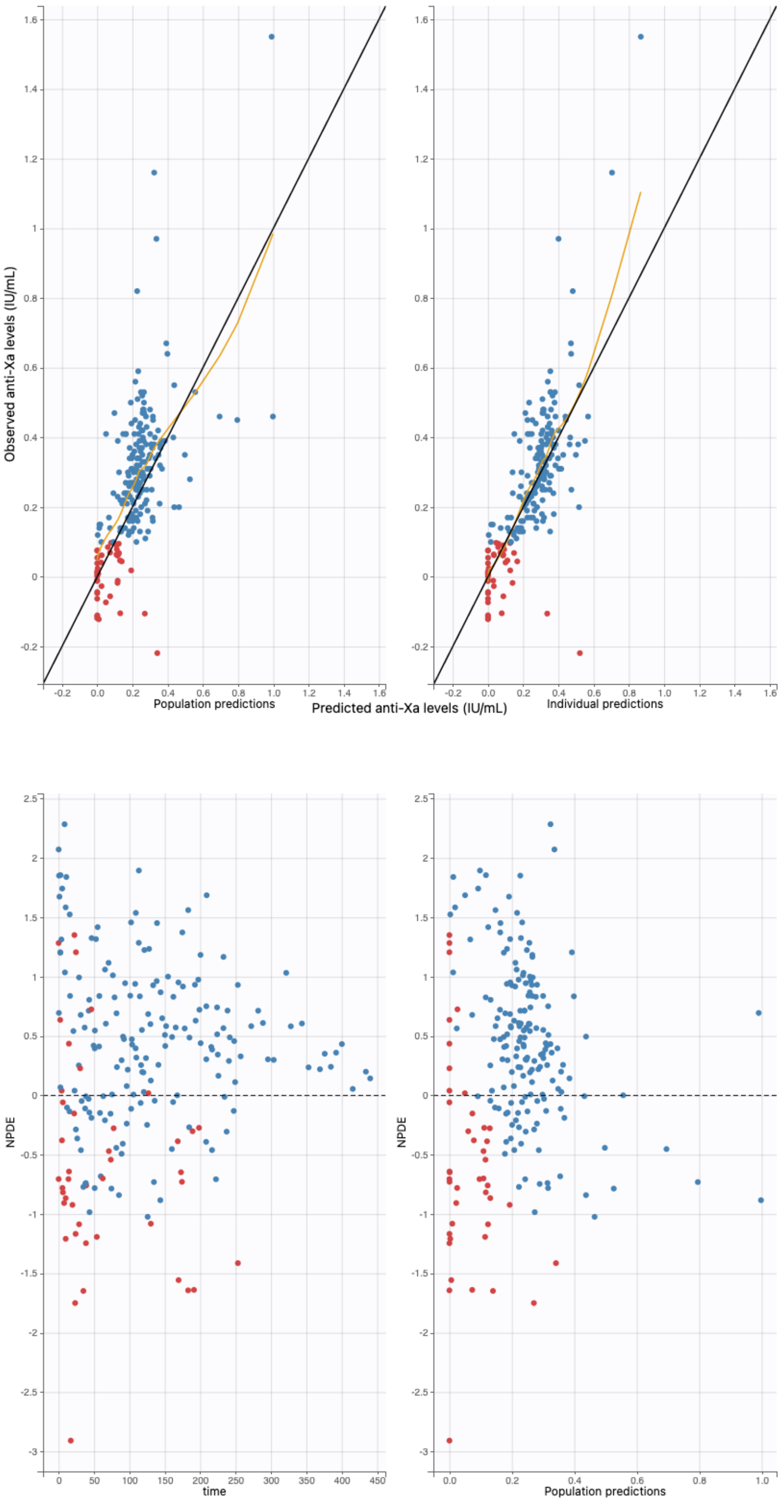

Figure S1. Goodness of fit plots for the validation cohort. Top panels: observations versus predictions. The black line represents the identity line. Blue circles represent the observed anti-Xa versus the corresponding predicted anti-Xa. Red circles represent the censored data. The yellow line represents the trend line. Left panel: plot of the observed anti-Xa (IU/mL) versus population predicted anti-Xa (no random component). Right panel: plot of the observed

anti-Xa versus individual predicted anti-Xa (with random component). Bottom panels: NPDE versus time and population predictions. The black line represents the identity line. NPDE, normalized prediction distribution errors.

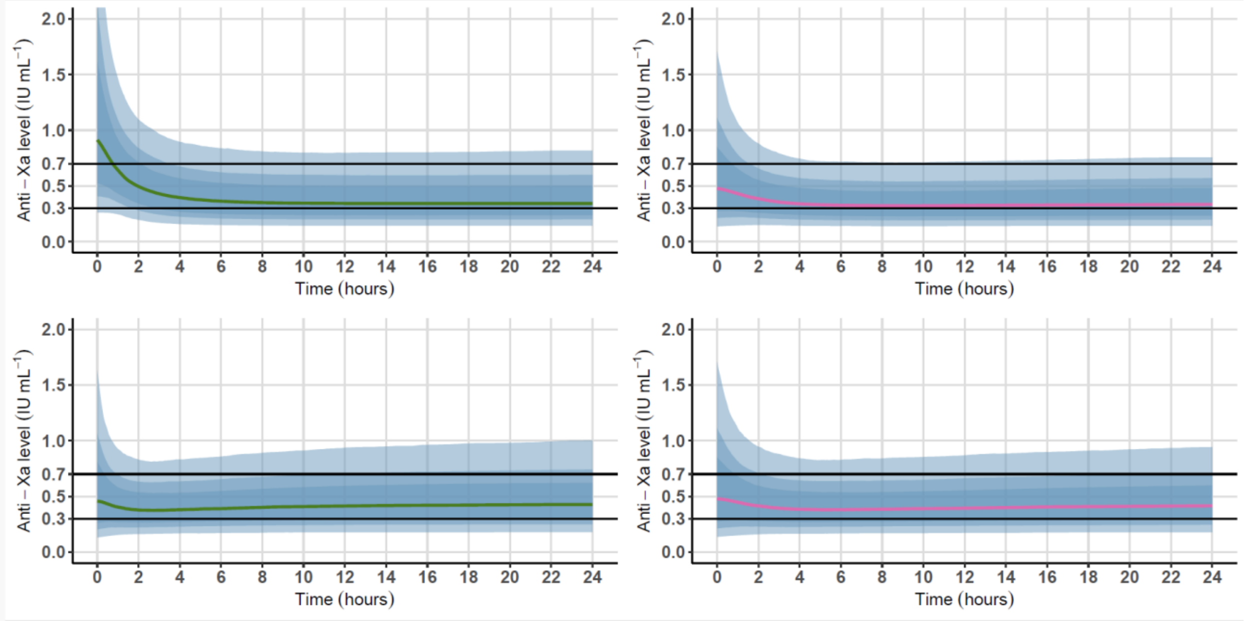

Figure S2. Simulations of the anti-Xa time courses using the final PK model with focusing on ECMO indication. Simulations were performed for a patient weighing 80 kg with a serum creatinine of 115 µmol/L and a CRP of 100 mg/L. Green line: Medical indication. Red line: Post cardiotomy indication. The black lines correspond to the recommended 0.3-0.7 IU/mL target anti-Xa interval. Blue shaded areas correspond to the interpatient variability intervals estimated in our model (50%, 70% and 90%, respectively). Top panels: simulations according to an 8000 IU (100 IU/kg) loading dose immediately followed by a 1200 IU/h (15 IU/kg/h) without adaptation to body weight, renal function, or CRP. Bottom panels: simulations according to our optimized dosing regimen. Bottom left panel: 4000 IU loading dose followed by a 1500 IU/h maintenance dose. Bottom right panel: 8000 IU loading dose followed by a 1500 IU/h maintenance dose.

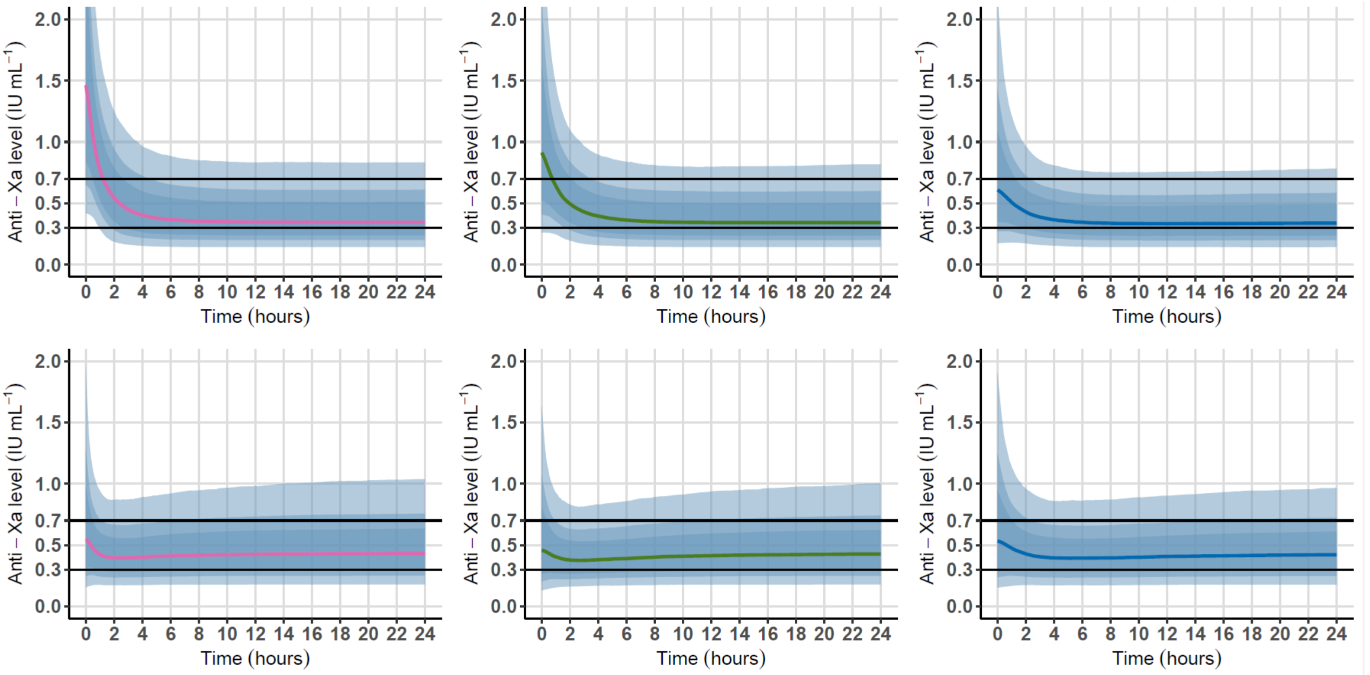

Figure S3. Simulations of the anti-Xa time courses using the final PK model with focusing on BW. Simulations were performed for a medical patient with a serum creatinine of 115 µmol/L and a CRP of 100 mg/L. Red line: 50 kg. Green line: 80 kg. Blue line: 120 kg. The black lines correspond to the recommended 0.3-0.7 IU/mL target anti-Xa interval. Blue shaded areas correspond to the interpatient variability intervals estimated in our model (50%, 70% and 90%, respectively). Top panels: simulations according to an 8000 IU (100 IU/kg) loading dose immediately followed by a 1200 IU/h (15 IU/kg/h) without adaptation to CRP or renal function. Bottom panels: simulations according to our optimized dosing regimen. Bottom left panel: 3000 IU loading dose followed by a 1500 IU/h maintenance dose. Bottom middle panel: 4000 IU loading dose followed by a 1500 IU/h maintenance dose. Bottom right panel: 7000 IU loading dose followed by a 1500 IU/h maintenance dose.

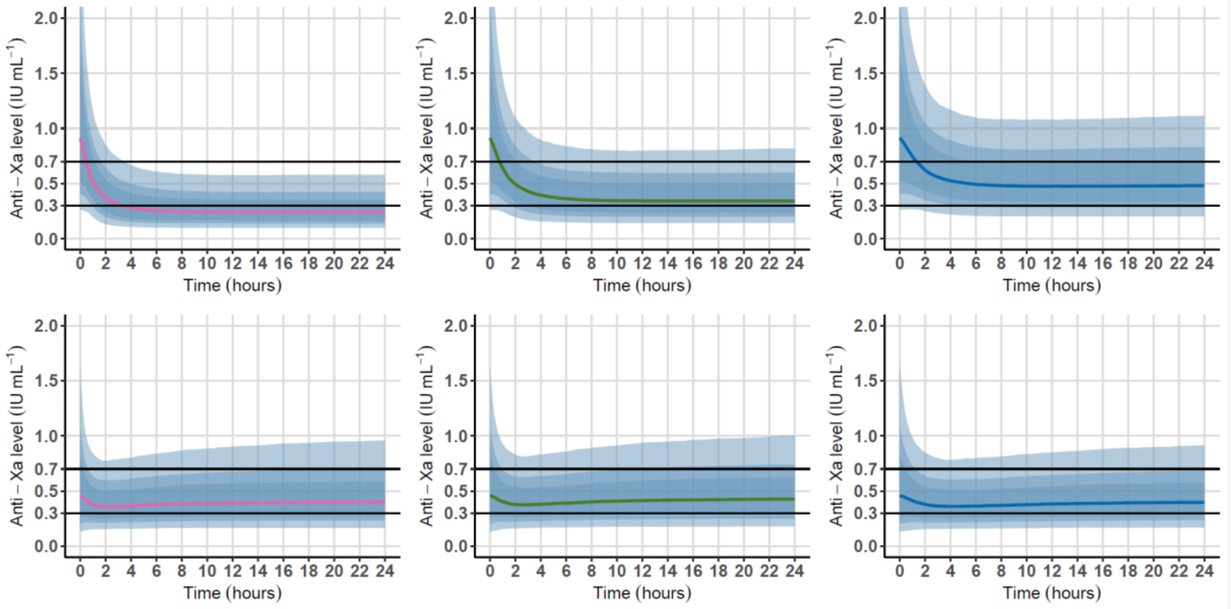

Figure S4. Simulations of the anti-Xa time courses using the final PK model with focusing on SCr. Simulations were performed for a medical patient weighing 80 kg with a CRP of 100 mg/L. Red line: SCr 25 µmol/L. Green line: SCr 115 µmol/L. Blue line: SCr 500 µmol/L. The black lines correspond to the recommended 0.3-0.7 IU/mL target anti-Xa interval. Blue shaded areas

correspond to the interpatient variability intervals estimated in our model (50%, 70% and 90%, respectively). Top panels: simulations according to an 8000 IU (100 IU/kg) loading dose immediately followed by a 1200 IU/h (15 IU/kg/h) without adaptation to body weight or CRP. Bottom panels: simulations according to our optimized dosing regimen. Bottom left panel: 4000 IU loading dose followed by a 2000 IU/h maintenance dose. Bottom middle panel: 4000 IU loading dose followed by a 1500 IU/h maintenance dose. Bottom right panel: 4000 IU loading dose followed by a 1000 IU/h maintenance dose.
